# Supplementary material for: Perirenal Fat Thickness Significantly Associated with Prognosis of Metastatic Renal Cell Cancer Patients Receiving Anti-VEGF Therapy
Source: Nutrients. 2022 Aug 18;14(16):3388. doi: 10.3390/nu14163388 (PMC9412489; doi:10.3390/nu14163388)
Supplement: Supplementary file 1 [file nutrients-14-03388-s001.zip › Table S1.pdf]

Table S1. Multivariate analysis of baseline characteristics predictive for overall survival and progression-free survival *.

Characteristics	Overall Survival **		Progression-free Survival **	
	HR (95%CI)	*p*-value	HR (95%CI)	*p*-value
Karnofsky score < 80 ***	1.92 (1.27-2.90)	0.002	1.62 (1.21-2.16)	0.001
Clear cell carcinoma	-	-	0.77 (0.59-1.00)	0.05
TDT < 1 year	1.87 (1.15-3.04)	0.01	1.01 (0.76-1.34)	0.93
Nephrectomy	-	-	0.85 (0.63-1.17)	0.34
Immunotherapy	0.51 (0.34-0.77)	0.001	-	-
Albumin < LLN, g/L	1.07 (0.71-1.60)	0.75	-	-
Hemoglobin < LLN, g/L	0.98 (0.63-1.53)	0.95	1.16 (0.87-1.56)	0.31
Corrected calcium > ULN, mmol/L	2.95 (1.68-5.19)	<0.001	1.83 (1.16-2.89)	0.01
PRFT > Median (1.6 cm) ****	0.57 (0.35-0.93)	0.01	0.78 (0.61-0.98)	0.04
SM > Median (128.9 cm^2)	-	-	0.81 (0.63-1.04)	0.10
VAT > Median (81.9 cm^2)	0.92 (0.46-1.84)	0.82	0.98 (0.65-1.49)	0.93
SAT > Median (100.2 cm^2)	0.65 (0.35-1.20)	0.17	0.98 (0.65-1.47)	0.93
TAT > Median (195.1 cm^2)	1.45 (0.62-3.43)	0.39	0.92 (0.53-1.58)	0.76

* Only variables that were significant in univariate analysis were included in multivariate analysis.

** 117 patients had died, and 267 patients had tumor progression.

*** Karnofsky score < 80 means Cancer patients cannot maintain a normal life and work.

**** Dichotomies of continuous variables was sex-specific in body composition.

BMI: body mass index; 95%CI: 95% confidence interval; HR: = hazard ratio;
LLN: lower limits of normal; PRFT: perirenal fat thickness; SAT : subcutaneous adipose tissue;
SM: skeletal muscle; SMI: skeletal muscle index; TAT: total adipose tissue; TDT: time from diagosis to treatment; ULN:upper limits of normal;VAT : visceral adipose tissue;
