# Supplementary material for: Perirenal Fat Thickness Significantly Associated with Prognosis of Metastatic Renal Cell Cancer Patients Receiving Anti-VEGF Therapy
Source: Nutrients. 2022 Aug 18;14(16):3388. doi: 10.3390/nu14163388 (PMC9412489; doi:10.3390/nu14163388)
Supplement: Supplementary file 1 [file nutrients-14-03388-s001.zip › TableS2.pdf]

Table S2. The c-index of IMDC expanded with body composition.

	Overall Survival		Progression-free Survival	
	C-index (95%CI)	*p*-value	C-index (95%CI)	*p*-value
IMDC *	0.69 (0.63-0.74)	$2.18x10^{-10}$	0.61 (0.57-0.65)	$1.50x10^{-6}$
Expanded with				
BMI	0.69 (0.64-0.75)	$3.95x10^{-10}$	0.62 (0.59-0.66)	$1.18x10^{-7}$
PRFT	0.71 (0.65-0.76)	$7.04x10^{-12}$	0.62 (0.58-0.66)	$3.83x10^{-7}$
SM	0.69 (0.64-0.74)	$2.73x10^{-10}$	0.62 (0.59-0.66)	$6.78x10^{-7}$
VAT	0.69 (0.64-0.75)	$1.47x10^{-10}$	0.62 (0.58-0.66)	$1.40x10^{-6}$
SAT	0.69 (0.63-0.74)	$1.44x10^{-10}$	0.62 (0.58-0.66)	$1.26x10^{-6}$
TAT	0.69 (0.64-0.74)	$2.03x10^{-10}$	0.62 (0.58-0.66)	$9.02x10^{-7}$
SMI	0.69 (0.64-0.74)	$7.20x10^{-10}$	0.61 (0.57-0.65)	$3.44x10^{-6}$
VAT/TAT	0.68 (0.63-0.74)	$7.39x10^{-10}$	0.62 (0.58-0.66)	$3.56x10^{-6}$

* IMDC model included time from diagosis to treatment of less than 1 year, Karnofsky performance status less than 80%, neutrophil greater than the upper limit of normal, platelets greater than the upper limit of normal, corrected serum calciumgreater than the upper limit of normal and serum hemoglobin less than the lower limit of normal.

BMI: body mass index; C-index: concordance index; 95%CI: 95% confidence interval

IMDC: International Metastatic Renal-Cell Carcinoma Database Consortium

SM: skeletal muscle; SMI: skeletal muscle index; TAT: total adipose tissue;

VAT : visceral adipose tissue
