# Supplementary figures and images for: Perirenal Fat Thickness Significantly Associated with Prognosis of Metastatic Renal Cell Cancer Patients Receiving Anti-VEGF Therapy
Source: Nutrients. 2022 Aug 18;14(16):3388. doi: 10.3390/nu14163388 (PMC9412489; doi:10.3390/nu14163388)

# Figure S1

## A

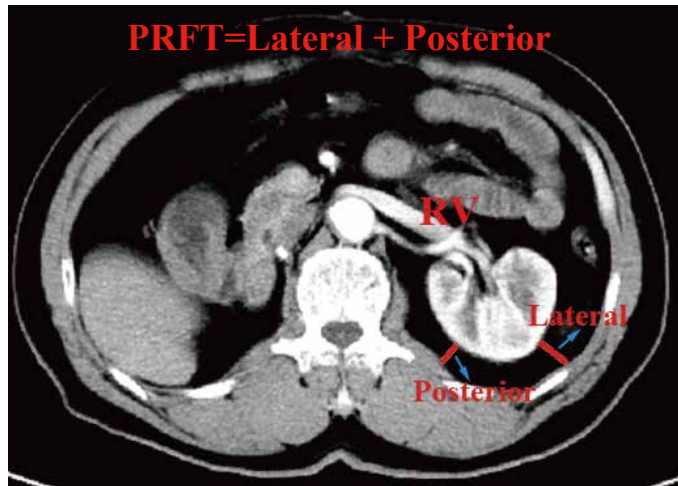

## B

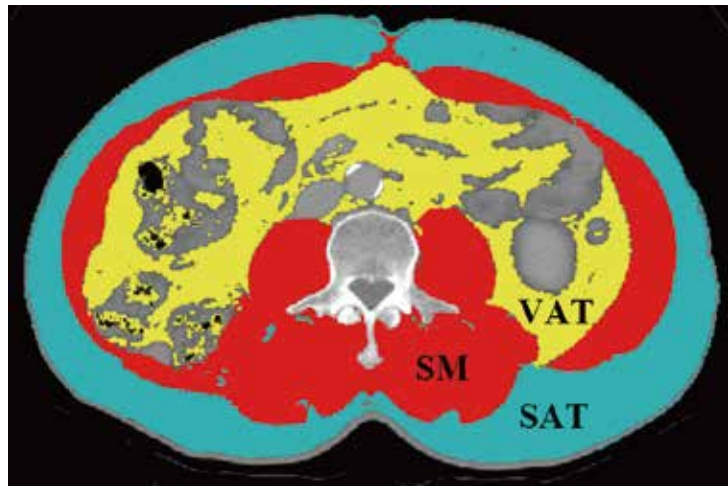

Supplement: Supplementary file 1 [file nutrients-14-03388-s001.zip › Figure S1.pdf]

**Figure S2**

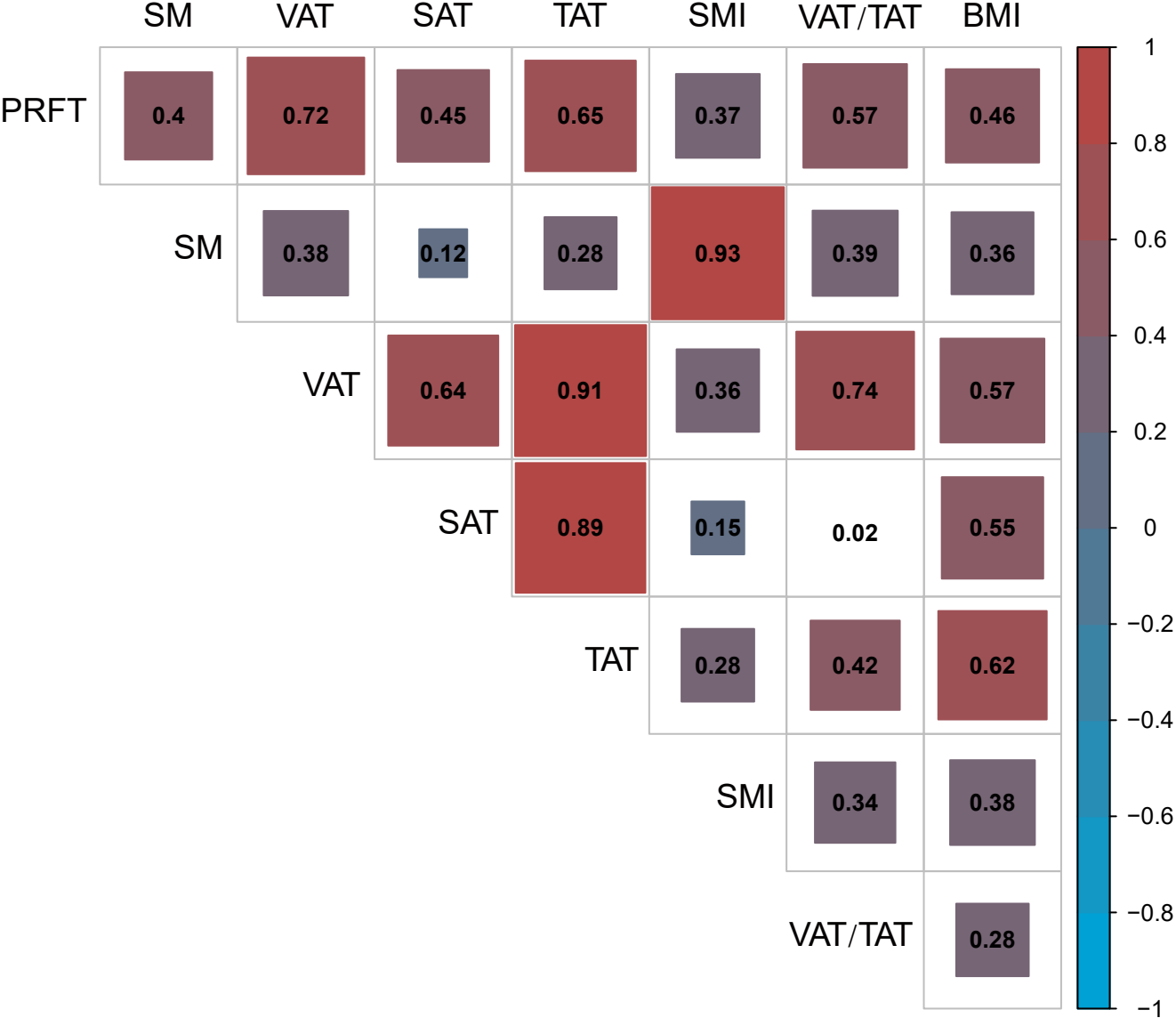

Supplement: Supplementary file 1 [file nutrients-14-03388-s001.zip › Figure S2.pdf]

Figure S3

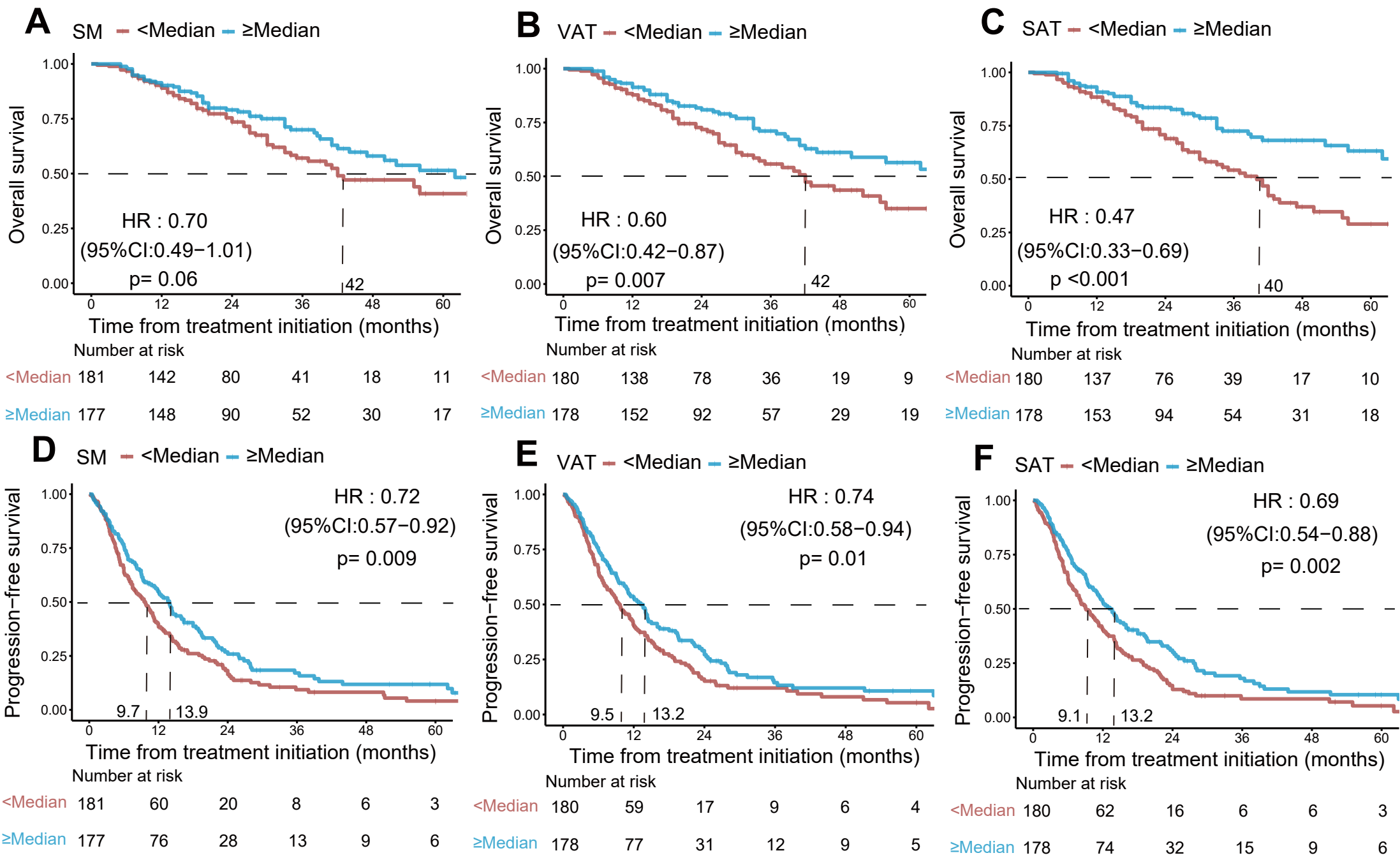

Supplement: Supplementary file 1 [file nutrients-14-03388-s001.zip › Figure S3.pdf]

Figure S4

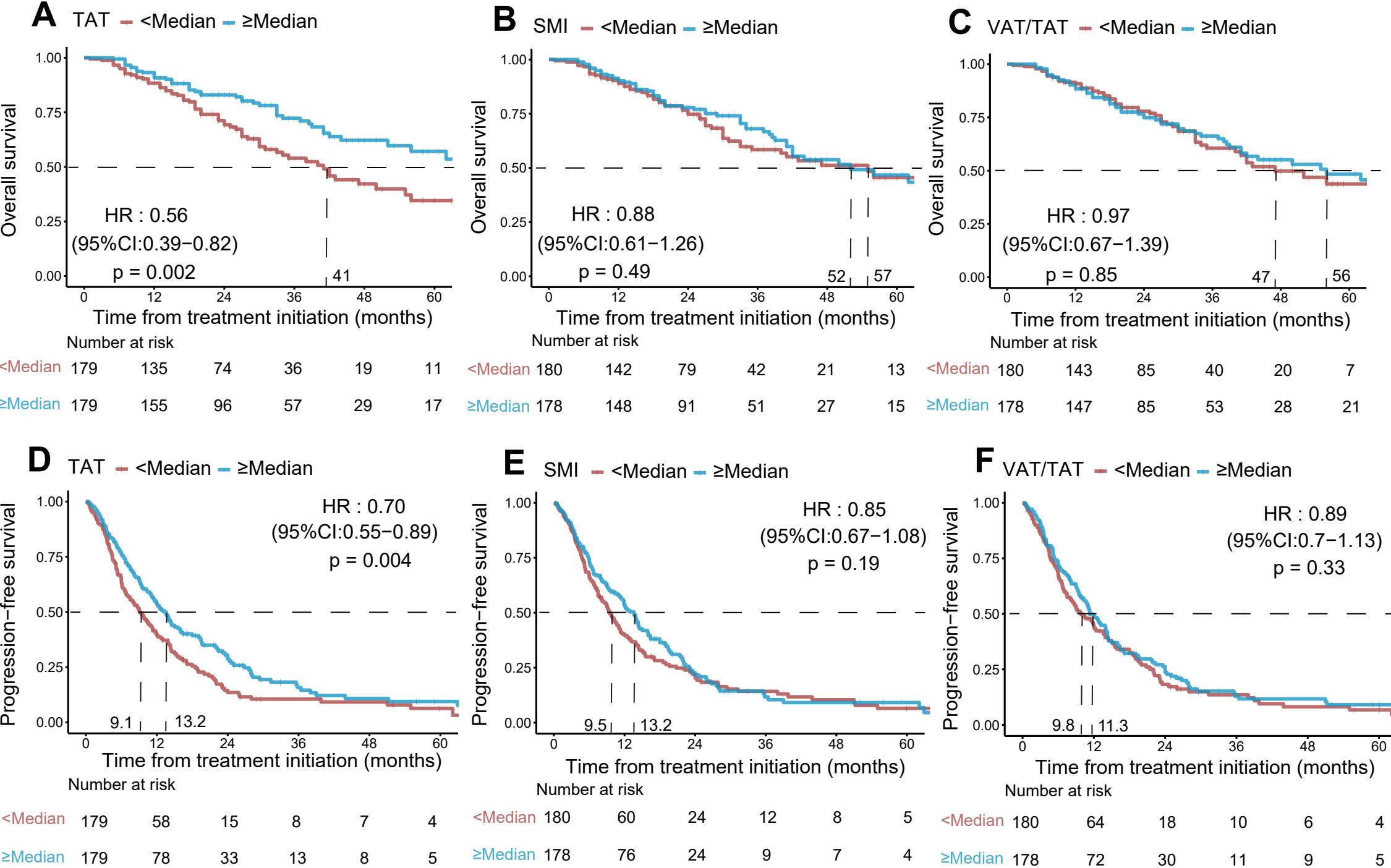

Supplement: Supplementary file 1 [file nutrients-14-03388-s001.zip › Figure S4.pdf]

Figure S5

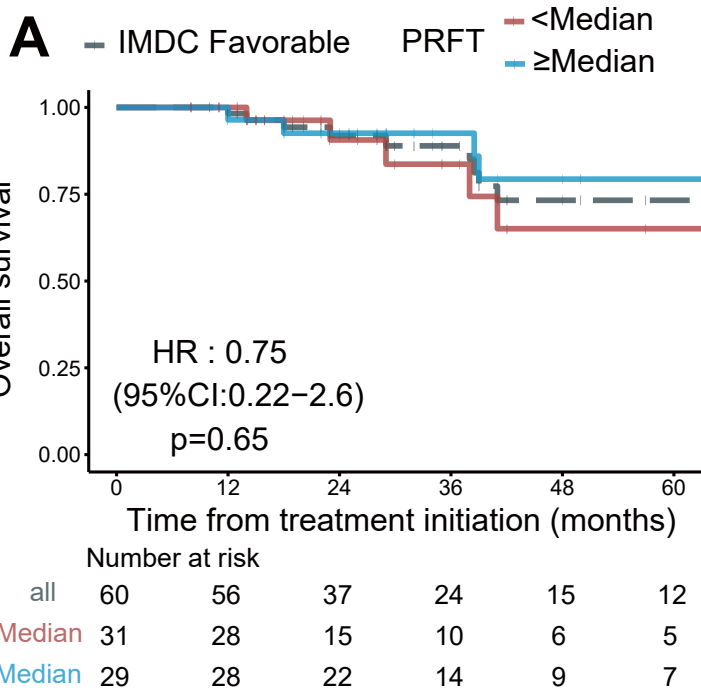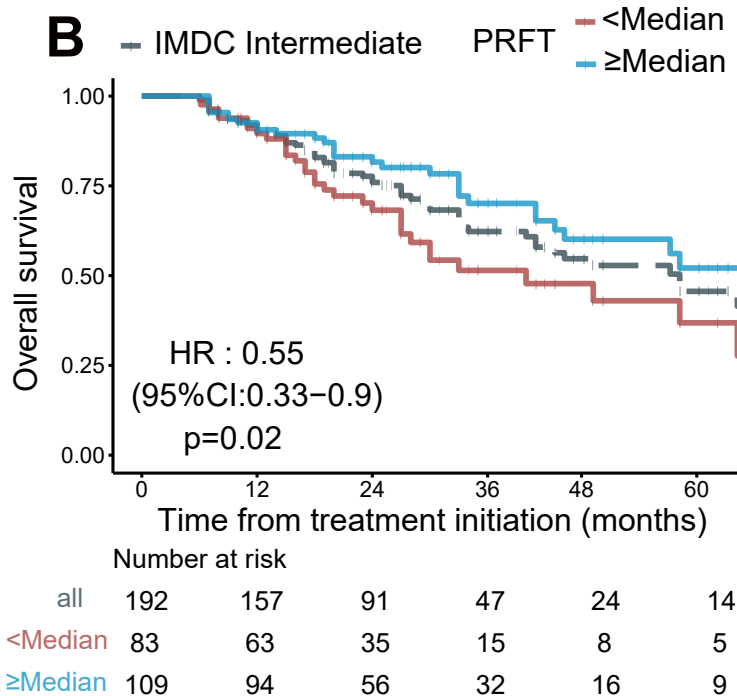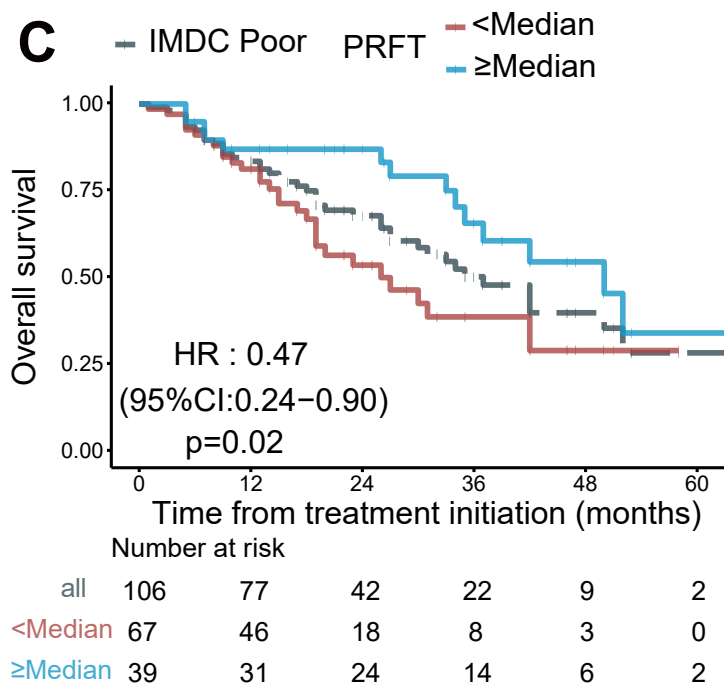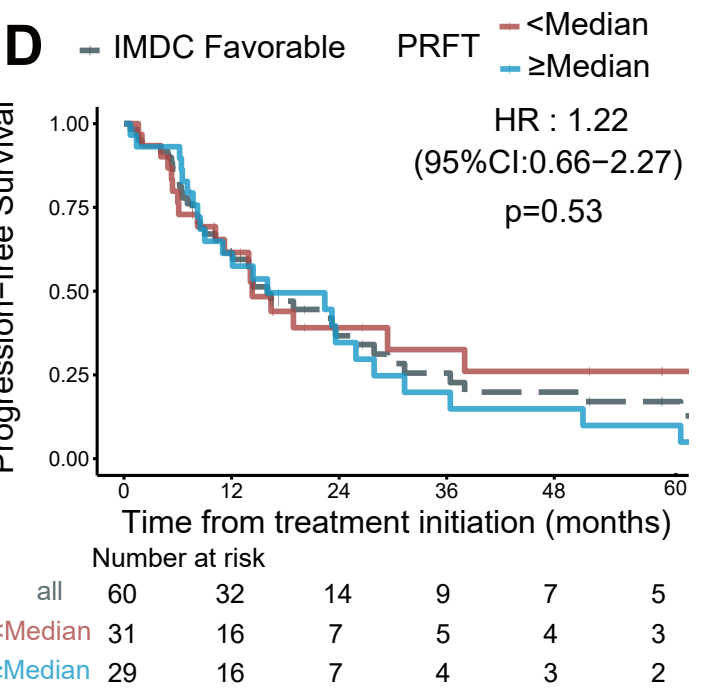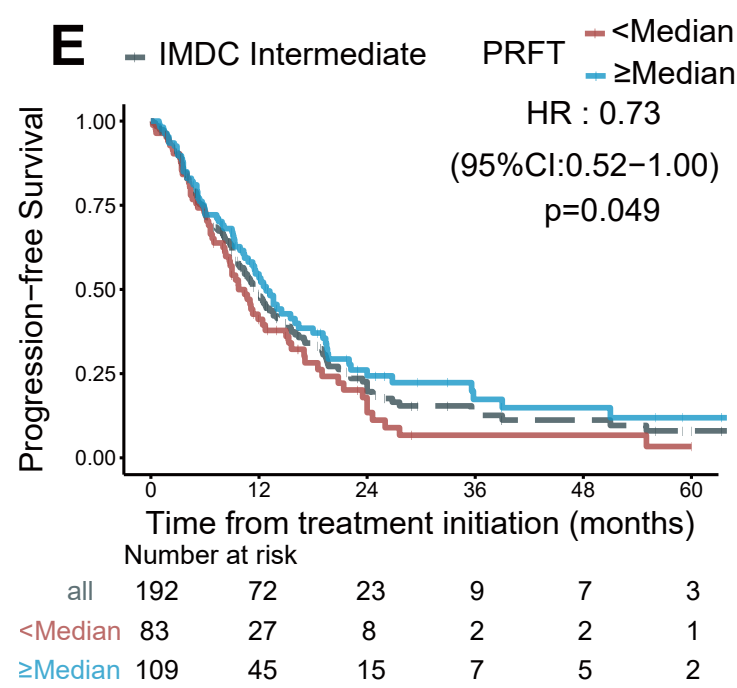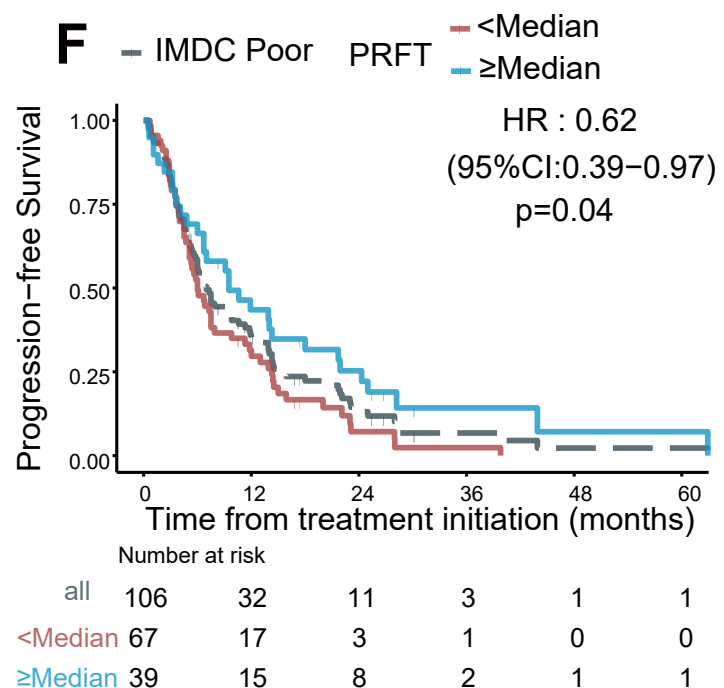

Supplement: Supplementary file 1 [file nutrients-14-03388-s001.zip › Figure S5.pdf]
